# Cultural competency and sensitivity in the curriculum for palliative care professionals: a survey in Switzerland

**DOI:** 10.1186/s12909-021-02745-1

**Published:** 2021-06-04

**Authors:** Orest Weber, Imane Semlali, Claudia Gamondi, Pascal Singy

**Affiliations:** 1 Liaison Psychiatry Service, Department of Psychiatry, Lausanne University Hospital, Lausanne, Switzerland; 2 Palliative and Supportive Care Clinic, Oncology Institute of Southern Switzerland, Bellinzona, Switzerland; 3 Liaison Psychiatry service, Av. de Beaumont 23, 1011 Lausanne, Switzerland

**Keywords:** cultural competence, cultural sensitivity, palliative care, cross-cultural training, communication

## Abstract

**Background:**

Cultural and linguistic diversity in patients and their relatives represents a challenge for clinical practice in palliative care around the world. Cross-cultural training for palliative care professionals is still scarce, and research can help determine and support the implementation of appropriate training. In Switzerland, health policies address diversity and equity issues, and there is a need for educational research on cross-cultural training in palliative care. The aim of this study was to investigate the clinical challenges faced by Swiss palliative care professionals when working with migrant patients and their relatives. We also documented professionals’ interests in cross-cultural training.

**Methods:**

A web survey of professionals working in specialized palliative care in the French- and Italian-speaking areas of Switzerland investigated clinical challenges with migrant populations and interests in various training opportunities.

**Results:**

A total of 204 individuals responded to the survey, 48.5 % of whom were nurses. The major difficulties they reported were communication impediments associated with patients’ linguistic and/or cultural backgrounds. In relation to educational needs, they expressed a particular interest in communication techniques that would allow them to deal with these issues autonomously. The professionals expressed less interest in training on collaborating with other professionals and examining one’s own stereotypes.

**Conclusions:**

Palliative care professionals’ post-graduate and continuing education must address communication techniques for sensitive palliative and end-of-life topics in cross-cultural contexts. Beginning with their pre-graduate studies, health professionals should assimilate the importance of collaborating with other professionals in complex cross-cultural situations and learn to reflect on their stereotypes and pre-conceptions in clinical practice.

## Background

In modern Western societies, many health professionals face communication challenges due to patients’ linguistic and cultural diversity [1]. Migration is a major contributor to this diversity [2]. Migrants may be defined as people who left their country voluntarily or involuntarily for economic, political, or personal reasons [3]. In palliative care, scientists have shown that language barriers and a lack of interpreters may complicate clinical interactions [4, 5]. Working with groups of migrants with limited knowledge about palliative care is especially challenging because of the association between palliative care and imminent death [4]. The common wish of close relatives to care for their relatives without any assistance by nurses adds complexity to interactions with many migrant families [6–8]. Written material in a variety of languages is needed but not always available [4].

European guidelines for palliative care stress the importance of training palliative care professionals in a culturally sensitive way [9, 10]. In many countries, public health authorities promote health equity measures in all domains of medicine, namely, in the form of cross-cultural training offerings [11, 12]. Such offerings allow health professionals to develop a variety of knowledge, skills, and attitudes to provide high-quality care to culturally diverse patients [13–17].

Switzerland has a high percentage of recent migrants. Among the country’s inhabitants, 25.1 % are foreigners [18], and 6.4 % do not speak a Swiss national language as their first language [19]. In urban centres, the percentage of foreigners ranges between 30 and 60 %. Nevertheless, pre- and post-graduate training offerings for all types of palliative care professionals rarely address issues related to linguistic and cultural diversity among patients [7]. Scientific knowledge is required to support the implementation of cross-cultural training in this clinical field, and such studies are of a high priority because current federal policies aim to support equity for migrants and other vulnerable groups in access to palliative care [20, 21].

A detailed picture of the main difficulties that palliative care professionals encounter in their work with migrants and of health professionals’ interests in various aspects of cross-cultural training is lacking for Switzerland and internationally. This kind of data will help educators anticipate their future audiences’ needs and reactions [22]. With our survey, we aimed to fill this gap by establishing (a) which difficulties working with migrant patients from linguistically and culturally diverse backgrounds professionals specialised in palliative care consider more important than others; (b) which aspects of cross-cultural competency or sensitivity training they consider more or less interesting; and (c) whether health professionals’ gender, age, profession and work setting affect their perceived difficulties in cross-cultural care and their training interests.

## Methods

### Study participants

 We conducted a web survey of all specialized palliative care professionals working in the French- and Italian-speaking areas of Switzerland. We included all professionals who had a clinical function with patients: physicians, nurses, psychologists, care assistants, spiritual assistants, social workers, etc. Secretaries, receptionists or other non-clinical professions were not included in this study.

### Study setting

Specialized palliative care in Switzerland encompasses four different settings of care [23]. Hospital palliative care units provide stationary care in public hospitals or other institutions (similar to the Hospice English model). Hospital mobile teams offer palliative care consultations in hospitals (e.g. oncology, internal medicine) and support other professionals in offering symptom control and psychosocial support across the continuum of life threatening disease. Home care mobile teams provide palliative care consultations at patients’ homes and in nursing homes. Outpatient facilities are commonly linked to large palliative care centres and their staff offer ambulatory consultations to palliative patients. These four settings of care were all represented in the study.

### Study questionnaire

The questionnaire was adapted from previous research conducted in oncology settings [22]. To ensure the applicability of the questionnaire in the palliative care context, we reviewed and adapted the questionnaire between March and June 2018, taking into account the literature and opinions of experts specializing in palliative care and cross-cultural communication. We modified items to integrate aspects related to end-of-life care (e.g., talking about death). Among the eight items we added, four were related to the management of patients’ cultural diversity related to beliefs, practices, and expectations towards end of life; three concerned the management of administrative and logistical tasks; and the last referred to colleagues’ attitudes towards diversity. Three former palliative care specialists, a physician, a nurse and a psychologist, with a vast amount of experience reviewed the questionnaire for face validity. They tested and approved its format and content. The questionnaire was first developed in French and then was forward and backward translated into Italian.

The questionnaire contained three parts. The first part investigated the frequency and intensity of difficulties faced by palliative care professionals when interacting with migrant patients and relatives. The initial question asked how often health professionals experienced communication difficulties related to linguistic and cultural diversity in migrant patients. The response format was a four-point scale, with 1 indicating *never*, 2 indicating *rarely*, 3 indicating *sometimes*, and 4 indicating *often*. The 21 items in this part of the questionnaire measured the degree to which difficulties in three domains were problematic. The first group of items was related to language barriers (Items 1, 16, and 18, see Table 1), the second group concerned the sociocultural background of migrant patients (Items 2, 3, 6, 7, 8, 11, 12, 14, 19, 20, 21, see Table 1), and the last group was related to lacking institutional resources and background knowledge (Items 4, 5, 9, 10, 13, 15, 17, see Table 1). Responses were collected on a four-point scale, with 1 indicating *not at all problematic*, 2 indicating *a little problematic*, 3 indicating *rather problematic* and 4 indicating *very problematic*.

**Table 1:** Difficulties in Clinical Interactions with Migrant Patients (*N* = 204)

	Not at all problematic		A little problematic		Rather problematic		Very problematic	
Variable	**N**	**%**	**N**	**%**	**N**	**%**	**N**	**%**
**Cluster I**								
1) Absence of a shared common language with the patient	3	1.5	23	11.3	87	42.6	91	44.6
2) Discussion of sensitive topics (e.g., end of life, death, intimacy)	10	4.9	35	17.2	98	48	61	29.9
3) Patients’ level of comprehension	7	3.4	40	19.6	105	51.5	52	25.5
4). Absence of written materials in other languages (e.g., brochures, consent forms)	12	5.9	35	17.2	91	44.6	66	32.4
5) Absence of referent individuals in cross-cultural clinical work	9	4.4	43	21.1	93	45.6	59	28.9
6) Patients’ perceptions of illness, death, treatments and the healthcare system (e.g., role of palliative care)	7	3.4	46	22.5	97	47.5	54	26.5
7) Patients’ financial resources (e.g., body repatriation, arrival of relatives)	14	6.9	46	22.5	85	41.7	59	28.9
8) Patients’ active involvement in decision making	18	8.8	43	21.1	95	46.6	48	23.5
**Cluster II**								
9) Lack of knowledge about living conditions and residence status of migrants	10	4.9	61	29.9	85	41.7	48	23.5
10) Lack of knowledge about health and support networks for precarious patients (e.g., asylum seekers, undocumented migrants)	13	6.4	60	29.4	86	42.2	45	22.1
11) Relatives’ involvement in care and decision making	17	8.3	56	27.5	94	46.1	37	18.1
12) Symptom and pain assessment and investigation of their meaning for patients	15	7.4	59	28.9	97	47.5	33	16.2
13) Access to professional interpreters	23	11.3	54	26.5	83	40.7	44	21.6
14) Integration of the diversity of religious and spiritual beliefs and practices (e.g., opinion about sedation)	21	10.3	60	29.4	82	40.2	41	20.1
15) Required efforts (e.g., time of consultation, organization)	23	11.3	60	29.4	108	52.9	13	6.4
16) Translation by relatives	32	15.7	65	31.9	79	38.7	28	13.7
**Cluster III**								
17) Logistics management (e.g., rooms for rituals, reception of important groups of visitors)	25	12.3	86	42.2	58	28.4	35	17.2
18) Collaboration with professional interpreters or colleagues providing translation	44	21.6	83	40.7	63	30.9	14	6.9
19) Colleagues’/superiors’ sensitivity to cultural diversity	32	15.7	96	47.1	56	27.5	20	9.8
20) Preconceived notions about certain groups of patients	48	23.5	100	49	49	24	7	3.4
21) Collaboration with religious representatives	53	26	103	50.5	43	21.1	5	2.5

The second part of the questionnaire asked about courses in cultural competency or sensitivity that the health professionals had already taken and aspects of training that they considered more or less interesting. This part started with a dichotomous yes-no question asking whether the health professionals had received any training in cultural competency or sensitivity. There were 17 items that measured the health professionals’ degree of interest in cross-cultural training. One group of items concerned aspects of training on communication issues (Items 7, 9, 16, see Table 2). The second group of items was centred on practical management of patients with different sociocultural backgrounds from one’s own (Items 1, 2, 3, 4, 6, 10, 11, 12, 14, 17, see Table 2). The last group of items referred to different types of background knowledge required for cross-cultural care (Items 5, 8, 13, 15, see Table 2). Responses were collected on a four-point scale, with 1 indicating *not at all interested*, 2 indicating *a little interested*, 3 indicating *rather interested* and 4 indicating *very interested*.

**Table 2:** Training Interests in Cross-Cultural Care (*N* = 204)

	Not at All interested		A little interested		Rather interested		Very interested	
Variable	**N**	**%**	**N**	**%**	**N**	**%**	**N**	**%**
**Cluster I**								
1) How to explore patients’ perceptions of illness, death, treatments and the healthcare system (e.g., role of palliative care)	2	1	13	6.4	46	22.5	143	70.1
2) How to discuss sensitive topics (e.g., end of life, death, intimacy)	6	2.9	17	8.3	50	24.5	131	64.2
**Cluster II**								
3) How to explore patients and relatives’ expectations	5	2.5	26	12.7	58	28.4	115	56.4
4) How to assess symptoms and pain and investigate their meaning for patients	10	4.9	25	12.3	55	27	114	55.9
5) Health and support networks for precarious patients (e.g., asylum seekers, undocumented migrants)	10	4.9	20	9.8	61	29.9	113	55.4
6) How to involve patients in decision making	7	3.4	27	13.2	58	28.4	112	54.9
7) How to adapt nonverbal communication	8	3.9	38	18.6	47	23	111	54.4
8) Inequality in access to care (e.g., socioeconomic, cultural, linguistic factors)	14	6.9	26	12.7	56	27.5	108	52.9
9) How to adapt speech to the patient’s level of comprehension	6	2.9	38	18.6	53	26	107	52.5
**Cluster III**								
10) How to break bad news	10	4.9	30	14.7	65	31.9	99	48.5
11) How to collaborate with relatives	7	3.4	38	18.6	64	31.4	95	46.6
12) How to cope with personal stereotypes and their impact on care	18	8.8	34	16.7	61	29.9	91	44.6
13) Historical and socioeconomic conditions of migrant populations in Switzerland	13	6.4	40	19.6	62	30.4	89	43.6
14) How to introduce religion and spirituality	12	5.9	38	18.6	66	32.4	88	43.1
15) Epidemiologic aspects of migration	18	8.8	36	17.6	64	31.4	86	42.2
16) How to collaborate efficiently with professional or nonprofessional interpreters	16	7.8	55	27	69	33.8	64	31.4
17) How to contribute to administrative tasks (e.g., visa application for relatives living abroad)	33	16.2	52	25.5	63	30.9	56	27.5

The third part of the questionnaire included sociodemographic items on the respondents’ gender, profession, work setting, age, number of years of work experience in health care (in total and in palliative care), country of birth, country in which the clinician was trained, number of languages for potential use in consultations, and first language.

### Data collection procedures

We used Limesurvey, an online survey software, to create and administrate the online questionnaire. The questionnaire format required the completion of all questions before the submission of the answers. Due to the lack of a Swiss database of palliative care professionals and institutions, we established an inventory of hospital units and clinics offering specialized palliative care by consulting the website Palliative.ch [24] and by contacting key actors in the field. We found 12 institutions in the targeted areas and reached all of them. Two responded negatively.

 In every palliative care facility, we asked service leaders to send us the email addresses of the staff members. Due to their limited time resources, service leaders were not able to specify how many persons of each profession were represented in these address lists. We sent the link to the online questionnaire to 74 health professionals from Riveneuve Foundation (Vaud), 69 from Lausanne University Hospital (Vaud), 17 from Aubonne Hospital (Vaud), 45 from Hospital of Northern Vaud, 43 from La Chrysalide (Neuchâtel), 27 from Jura Hospital, 90 from Geneva University Hospital, 41 from Tara House (Geneva), 23 from Freiburg Hospital, and 26 from Bellinzona and Valeys Regional Hospital (Ticino). In total, 455 health professionals were reached.

The survey was open from June to August 2018, and all the health professionals received two email reminders, 2 and 4 weeks after the initial invitation.

### Data analyses

A statistical analysis of the results was conducted. Because of the nominal nature of the data, the researchers selected two descriptive methods: frequency analysis and comparative analysis We clustered answers regarding difficulty and interest according to their frequency, using a procedure followed in Weber et al. [22]. In this process, we establish cut-off values which allow us to group items into clusters of comparable size. For this reason, the clustering process is presented among the results of the study. We used an ordered dependent variable model, using the F-test in Eviews (edition 9.5), to check the statistical significance of the relationship between health professionals’ sociodemographic characteristics and their responses to the survey questions. The variables with a 0.05 level of statistical significance were considered for additional analysis.

## Results

### Sample

We received 204 complete questionnaires, for a response rate of 45 %. Table 3 presents the respondents’ sociodemographic characteristics. Most respondents were women (80.9 %), and nearly half of the respondents were nurses (48.5 %). Most respondents were from inpatient units in hospitals. Almost half of the respondents were foreign-born (46.1 %). Three-quarters of the respondents worked in the cantons of Vaud and Geneva, and nine respondents worked in the canton of Ticino. The distribution of the respondents among these cantons reflects the demographic distribution between French- and Italian-speaking cantons.

**Table 3:** Sociodemographic characteristics of the respondents

Characteristics	Values	N	%
**Gender**	Female	165	80.9
	Male	39	19.1
**Profession**	Nurse	99	48.5
	Physician	33	16.2
	Care assistant	14	6.9
	ASSC (community health and care assistant)	10	4.9
	Volunteer	9	4.4
	Psychologist	9	4.4
	Spiritual assistant (chaplain)	6	2.9
	Social worker	6	2.9
	Physiotherapist	6	2.9
	Occupational therapist	4	2
	Nutritionist	3	1.5
	Other^1^	5	2.5
**First language**	French	161	78.9
	Portuguese	16	7.8
	German	12	5.9
	Italian	4	2
	Other^2^	11	5.4
**Country of birth**	Switzerland	110	53.9
	France	53	26
	Italy	6	2.9
	Other^3^	35	17.2
**Country of training**	Switzerland	134	65.7
	France	42	20.6
	Italy	6	2.9
	Other^4^	22	10.6
**Work setting**	Hospital palliative care units	137	67.2
	Hospital mobile teams	38	18.6
	Home care mobile teams	35	17.2
	Outpatient facilities	12	5.9
	Other^5^	3	5.4
**Cantons**	Vaud	92	45.1
	Geneva	61	29.9
	Neuchâtel	17	8.3
	Jura	15	7.4
	Freiburg	9	4.4
	Ticino	9	4.4
	Bern	1	0.5

^1^Art therapist, Mediator, Dietician; ^2^Arabic, Spanish, Dutch, English; ^3^Tunisia, Belgium, Ivory Coast, Germany, Spain, Netherland, Portugal, USA, Brazil, Canada, Cameroon, Mozambique, Sweden, Argentina, Senegal, Guinea, Chili, Syria; ^4^Portugal, Tunisia, Belgium, Spain, Argentina, Canada, Germany; ^5^Independent nurses

### Challenges in clinical communication with migrant patients

More than 80 % of the health professionals reported that they sometimes or often had difficulties communicating with migrant patients. Only 1 % reported never having faced such challenges.

Table 1 presents all difficulties communicating with migrant patients listed in the questionnaire by degree of problematicity. We divided the 21 items into three clusters based on problematicity. For Cluster I, more than 70 % of health professionals considered the scenarios described in the items to be rather or very problematic. Cluster II comprises items with rates of rather problematic and very problematic responses between 50 and 70 %, and Cluster III comprises items with rates of rather problematic and very problematic responses of less than 50 %.

For Cluster I, we identified eight highly problematic items. The most prominent were the absence of a shared common language, discussions of sensitive topics, patients’ level of understanding, and the absence of both written material in other languages and referent individuals in cross-cultural clinical work. Cluster I also contained items referring to difficulties related to patients’ perceptions, financial resources, and active involvement in decision-making.

Eight items were assigned to Cluster II. Health professionals’ lack of knowledge about migrants’ living conditions in Switzerland (including residence documents and status) and about health and support networks were categorized in this cluster. This cluster also included items on difficulties with involving relatives, assessing symptoms and pain, and integrating religious and spiritual diversity into treatment. Access to interpreters, translation by relatives, and the efforts required by such clinical work were the three remaining items in this cluster.

Cluster III, as the least problematic cluster, contained items on five aspects: logistics management, collaboration with interpreters and/or translators, colleagues’ or superiors’ sensitivity to cultural diversity, health professionals’ own preconceptions, and collaboration with religious representatives.

Our statistical analyses showed a significant impact of the respondents’ social and biographical characteristics on their answers related to perceived difficulties in clinical work with migrant patients (*p* ≤ 0.05, see Table 4). Respondents who reported having taken courses in cross-cultural care found that the absence of referent individuals in cross-cultural care was less problematic than did other health professionals (*p* = 0.005). More women than men in the sample considered patients’ limited financial resources to be a difficulty of working with vulnerable migrants (*p* = 0.039). Being a physician (*p* = 0.000), being born in Switzerland (*p* = 0.008), and working in hospital mobile teams (*p* = 0.006) or home care mobile teams (*p* = 0.02) were related to considering translation by the patient’s relatives to be problematic. Belonging to a hospital unit, in contrast, was associated with considering translation by the patient’s relatives to be less of a problem (*p* = 0.001).

**Table 4:** Statistical significance of perceived difficulties with sociodemographic variables

	Gender	Age	Profession	Country of birth	Canton	Hospital unit	Hospital mobile team	Home care mobile team	Outpatient facilities	Previous training
Variable	**P**	**p**	**p**	**p**	**P**	**p**	**p**	**P**	**p**	**p**
**Cluster I**										
4) Absence of written materials in other languages (e.g., brochures, consent forms)		0.044								
5) Absence of referent individuals in cross-cultural clinical work										0.005
6) Patients’ perceptions of illness, death, treatments and the healthcare system (e.g., role of palliative care)						0.006				
7) Patient’s financial resources (e.g., body repatriation, arrival of relatives)	0.039									
8) Patients’ active involvement in decision making									0.01	
**Cluster II**										
10) Lack of knowledge about health and support networks for precarious patients (e.g., asylum seekers, undocumented migrants)					0.031					
15) Required efforts (e.g., time of consultation, organization)									0.004	
16) Translation by relatives			0.000	0.008	0.009	0.001	0.006	0.02		
**Cluster III**										
18) Collaboration with professional interpreters or colleagues providing translation								0.05		
19) Colleagues/superiors’ sensitivity to cultural diversity				0.022						

### Interests in cultural competency and sensitivity training

Among the respondents, 67.6 % reported having never received specific training on cross-cultural topics. Table 2 depicts the distribution of responses according to degree of interest in each aspect of training listed in the questionnaire. The vast majority of respondents reported being rather or very interested in all aspects of training. Considering the generally high interest, we focused on the percentage of respondents who were very interested when clustering the items into three groups. For Cluster I, more than 60 % of health professionals were very interested in the training aspects described in the items. Cluster II comprises items with rates of very interested responses between 50 and 60 %, and Cluster III comprises items with rates of very interested responses of less than 50 %.

Cluster I contained only two aspects of training that triggered particularly strong interest among health professionals. More than 70 % of respondents were very interested in courses on how to explore patients’ perceptions of illness, death, treatments, and the healthcare system. Their interest was slightly lower but still very high for training on how to improve discussions of sensitive topics with culturally diverse patients.

The seven items in Cluster II focused mainly on procedural skills oriented towards exploring expectations, assessing symptoms and pain, promoting active involvement in decision-making, and adapting nonverbal communication and speech to patients. The two knowledge items in this cluster related to migrants’ local health and support networks and factors of inequality in access to care.

In Cluster III, eight items triggered slightly lower interest among health professionals. Six items referred to procedural skills: breaking bad news, introducing religion or spirituality, coping with stereotypes, contributing to administrative tasks, and collaborating with relatives and professional or nonprofessional interpreters. There were also two topics related to general knowledge among these items, namely, on the historical and socioeconomic conditions of migrants and epidemiologic aspects of migration.

We found significant sociodemographic differences in the responses about training interests in 17 cases (see Table 5). Women (*p* = 0.026) and nurses (*p* = 0.007) were more interested in learning how to better explore patients’ perceptions than men and other professionals, respectively. Women also expressed more interest than men in receiving information about health and support networks for precarious patients (*p* = 0.017). Care assistants reported adapting terminology to patients’ comprehension levels to be more important than did other professionals (*p* = 0.021), whereas both nurses and care assistants indicated that optimizing the assessment of symptoms and pain was more essential than did other professionals (*p* = 0.027). Finally, health professionals working in the canton of Ticino (Italian-speaking Switzerland) showed the strongest interest in courses on interactions with relatives (*p* = 0.009).

**Table 5:** Statistical significance of training interests by sociodemographic variables

	Gender	Age	Profession	Country of birth	Canton	Home care mobile teams	Previous training
Variable	**p**	**p**	**p**	**p**	**p**	**p**	**p**
**Cluster I**							
1) How to explore patients’ perceptions of illness, death, treatments and the healthcare system (e.g., role of palliative care)	0.026		0.007				
2) How to discuss sensitive topics (e.g., end of life, death, intimacy)					0.000		
**Cluster II**							
3) How to explore patients and relatives’ expectations						0.002	
4) How to assess symptoms and pain and investigate their meaning for patients			0.027	0.012	0.000		
5) Health and support networks for precarious patients (e.g., asylum seekers, undocumented migrants)	0.017						
6) How to involve patients in decision making					0.007		
9) How to adapt speech to patient’s level of comprehension			0.021				0.026
**Cluster III**							
10) How to break bad news		0.031			0.03		0.017
11) How to collaborate with relatives					0.009		
13) Historical and socioeconomic conditions of migrant populations in Switzerland							0.026
15) Epidemiologic aspects of migration				0.031			

## Discussion

### Main results in light of the literature

Our survey produced original findings on Swiss palliative care professionals’ experiences with migrant patients and their training needs and interests related to cross-cultural care. They perceived interactions with migrant patients and their relatives to be difficult. The palliative care professionals identified language barriers, sensitive topics (e.g., end-of-life, death, and intimacy) and patients’ comprehension of illness and the healthcare system (e.g., role of palliative care) as major challenges in these interactions. In comparison, collaborating with other professionals (e.g., interpreters) and relatives was reported to be rather unproblematic, as was examining one’s own stereotypes. A vast majority of the respondents also reported a high level of interest in cross-cultural training. Their priorities were in line with their answers on difficulties in cross-cultural clinical interactions. Courses on techniques to provide access to migrant patients’ lifeworld and priorities (e.g., perceptions of illness and death, expectations, and meaning of symptoms) were reported to be among the most important needs. Learning how to collaborate with others and learning how to examine stereotypes were lower priorities.

Comparable surveys from other countries and in other medical specialties have yielded similar results regarding the difficulties that health professionals experience in their practice with migrant patients. Communication barriers and patients’ cultural particularities have been rated as the most important factors in all these studies [12, 22, 25]. The scores indicating the respondents’ interests in specific areas of cross-cultural training also show similarities with those of a former study in oncology [22]. Health professionals working in specialized palliative care thus do not seem to differ significantly from colleagues working in other domains.

Patient-centred communication is crucial for high-quality clinical work with culturally and linguistically diverse patients [26]. It seems logical that experienced palliative care professionals would focus on this aspect. However, this focus may also signify a strong wish to deal with complex clinical situations autonomously. This hypothesis is reinforced by the respondents’ comparatively limited interest in enhancing collaboration with non-clinical professionals such as interpreters. Some forms of certainty regarding culture – rather than of cultural sensitivity or humility – may thus exist in palliative care (17). According to conceptual and scientific work on cross-cultural teaching and care, this certainty threatens equity in care, whereas cultural sensitivity and humility support equity [17, 27]. The ways in which cultural competence and sensitivity are taught in the curricula of health professionals who ultimately work in palliative care may thus influence access to health resources during terminal illness and at the end of life.

### Trends in sociodemographic variation within the sample

The statistically significant relations between participants’ sociodemographic characteristics and their responses indicate differences between subgroups in the study. Some significant differences triggered explanatory hypotheses, based either on the existing literature on subgroups of the study population or the research team’s insider knowledge of the field.

The well-documented trend of women placing more focus than men on social aspects in clinical work [28, 29] likely accounts for most of the variation in responses according to gender. Indeed, women appeared to express more dilemmas in clinical work related to patients’ financial resources and expressed a stronger interest in learning how to investigate patients’ representations and learning about migrants’ support networks. However, nurses’ professional culture may play a role, as the survey showed that 73 % of women respondents were nurses. Nurses may differ from physicians in their interpretation of the priority to “care” in comparison with physicians’ priority to “cure” [30]. A major difference between Italian-speaking Switzerland (Ticino) and French-speaking Switzerland lay in the higher interest in courses on collaboration with relatives among the respondents from Ticino. This finding may be related to the fact that professionals and patients in Ticino may hold a value system marked by more familism (Mediterranean culture) than the rest of Switzerland [31].

Palliative care physicians perceived the practice of using relatives as translators to be problematic. Since physicians in Swiss hospitals are responsible for treatment decisions and the avoidance of medical errors, the high risk of miscommunication associated with this practice may explain this trend. Translation by relatives was reported to be more problematic by mobile hospital teams than by other hospital staff. This finding may be partially explained by the fact that mobile teams have less access to professional interpreters and may be more aware of the negative consequences of nonprofessional interpreting. Our finding that care assistants were particularly interested in learning to adapt their wording to patients’ levels of comprehension may be explained by their intensive contact with migrant patients and relatives with all kinds of linguistic profiles and without any help from professional interpreters. All these profession- and setting-related trends should remind training organizers and teachers that the perceived practical and relational impacts of language barriers and language interpretation resources may vary significantly within audiences and that courses should be able to address this adequately.

### Practical implications

Overall, our research provides evidence of the importance of cross-cultural training [see [32–35] for comparable findings in other countries]. The results also provide practical ideas for educational offerings in the pre-graduate, post-graduate and continuing education of health professionals who will work in palliative care.

Pre-graduate clinical curricula usually include generic courses on patient-centred communication. These courses are apparently not sufficient to cover palliative care professionals’ needs regarding working with migrants. Educators in charge of these generic courses might need to strive towards cultural mainstreaming in their training offerings. They may, for instance, use more case reports describing and standardized patients belonging to cultural, socioeconomic and linguistic minorities [36]. Pre-graduate curricula should also encompass knowledge on culture, socioeconomic disparities, language barriers, key external partners (interpreters, chaplains, etc.) and stereotypes [12, 37] in a way that creates cultural sensitivity and counters excessive certainties regarding culture. According to our results, teachers might need to use more arguments and effort to convince male participants of certain information, especially regarding the importance of patients’ socioeconomic circumstances for clinical work.

Post-graduate and continuing education in palliative care should focus on procedural skills for culturally sensitive history taking and treatment planning with end-of-life patients and consolidate reflexivity on stereotypes and collaborations. In our experience, an optimal approach consists of case presentations followed by peer and expert advice on alternative anamnestic questions and alternative means of discussion with patients and families. Kleinman’s questions for the exploration of the patient’s explanatory models of illness [38], questions from the cultural formulation of the DSM [39] and the chapter on migrants of the Calgary-Cambridge manual [40] contain key resources for teachers. The presence of a psychologist or psychiatrist among experts is helpful since emotional aspects (e.g., powerlessness and defence mechanisms) are often entangled with cultural and communicational aspects. The application of learned content by groups of physicians between training sessions is an ideal educational method.

### Limitations and research perspectives

The comparatively high survey response rate is a strength of this study [12, 22, 41], but the study also has some limitations. A comparison between the data from the sample of respondents with data on the total population of professionals working in specialized palliative care would have enriched the interpretation of the data [see 22 for similar limitations]. Furthermore, our sample does not reflect the overall opinions and experiences of palliative care professionals in Switzerland since the German-speaking cantons were not included in the study. The limited resources of the project did not allow translation of the questionnaire into German and its distribution in German-speaking health structures. Moreover, health professionals with stronger interest in migration and diversity may have been particularly keen to participate in this survey, and interest in cross-cultural training may thus have been overrated [42]. Equally, the respondents had to complete the whole questionnaire before submitting it, in order to avoid missing answers. Study participants did therefore not have the option not to answer any question and this may have led to the drop out of some of the less motivated participants.

The survey should be extended to all of Switzerland. Qualitative studies are needed to elucidate unexplained patterns of socio-biographic variation in the responses. In addition, our research team already carried out a qualitative focus group study with the goal of addressing the abovementioned practice implications based on the opinions of groups of experts and educators in cross-cultural and palliative care.

## Conclusions

In this survey, Swiss palliative care professionals reported significant difficulties in their work with migrant patients as well as major interest in cross-cultural training. Educational offerings should be developed and adjusted with regard to these difficulties and interests. Coherent concepts of cross-cultural training for all professionals involved in palliative and end-of-life care should allow health professionals to acquire useful communication tools and key skills related to cultural sensitivity and humility, such as the ability to recognize their limitations, to seek help and to reflect on biases and stereotypes [20]. These skills should be taught at the pre-graduate level and reinforced in practice-based courses during post-graduate and continuing education to prevent excessive certainties regarding culture in health professionals.

## Data Availability

The datasets generated and/or analysed during the current study are not publicly available due to technical reasons but are available from the corresponding author on reasonable request.
